# Measuring Burnout Among Psychiatric Residents Using the Oldenburg Burnout Inventory (OLBI) Instrument

**DOI:** 10.25122/jml-2019-0089

**Published:** 2019

**Authors:** Raluca Oana Tipa, Catalina Tudose, Victor Lorin Pucarea

**Affiliations:** 1. Department of Psychiatry, “Prof. Dr. Alexandru Obregia” Clinical Hospital of Psychiatry, Bucharest, Romania; “Carol Davila” University of Medicine and Pharmacy, Bucharest, Romania; 2. Department of Marketing and Medical Technology, “Carol Davila” University of Medicine and Pharmacy, Bucharest, Romania

**Keywords:** psychiatric residents, burnout, exhaustion, disengagement, mental health

## Abstract

Burnout is prevalent among mental health providers and is significantly associated with the employee, consumer, and organizational costs. Nowadays, burnout prevalence is increasing and can challenge the residents’ professional development, place patients at risk, and have a significant influence on a variety of personal costs.

Considering its importance, this research attempted to measure the burnout experienced by Romanian psychiatric residents while also correlating demographic characteristics and work situations.

A cross-sectional study was conducted on 116 Romanian psychiatric residents. Our questionnaire contained socio-demographic information and burnout assessment, which was performed using the Oldenburg Burnout Inventory (OLBI).

The burnout scores were classified as high burnout (22.4% of the respondents), moderate burnout (51,7% of the respondents), and low burnout (25.9% of the respondents). As such, all psychiatric residents who suffered from high levels of burnout were satisfied with their salary and their work but dissatisfied with the resources available for attending patients.

From all physicians who might experience burnout, psychiatrists are most likely to search for help. The fact that the majority of psychiatric residents in our study were satisfied with their salary and their work, but dissatisfied with the available resources for attending patients might be a result of the Romanian policy of increasing incomes for medical personnel.

In conclusion, adding stress management training to the medical education curriculum could help the residents to deal more effectively with the training strain, develop personal techniques for helping themselves to improve their professional path, and potentially prevent upcoming physician burnout.

## Introduction

Burnout has become globally recognized as a significant public health issue [1]. In the past 35 years, plentiful intervention studies have been conducted [2] and we found abundant research on burnout associated with stress and satisfaction [3-6], with mental health consequences [7-10] and furthermore with internal marketing as an instrument which can improve burnout symptoms [11] by helping in educating, stimulating, guiding and leading workforce to higher levels of performance and gratification [12].

Burnout prevalence is increasing and also affects medical students, residents, and more than half of all practicing physicians [13]. More than that, burnout has reached epidemic levels, with a prevalence near or exceeding 50%, as documented in national studies of both physicians in training and practicing physicians [14]. A systematic review found that there are relatively high rates of burnout amongst medical students, residents in training, and physicians ranging from 7 to 80%, although the rates may vary according to discipline [15]. Regarding psychiatric residents, another systematic review found that the overall burnout rate was 33.7% [15].

Besides burnout, physicians are very vulnerable, being prone to stress, suicidal ideation, and even suicide per se [16, 17]. Additionally, high levels of stress for healthcare professionals have been linked to diminished work efficiency and high rates of staff turnover [18].

Moreover, burnout is also associated with anxiety, depression, and substance use [10, 17, 19]. Burnout is prevalent among mental health providers and is significantly associated with the employee, consumer, and organizational costs [2]. Out of all medical areas, psychiatrists have the most intense form of interpersonal contact with patients, regularly dealing with different kinds of “difficult” patients [20]. Mental health is, in many ways, a unique domain. In addition to the problematic nature of providing mental health services, the segment is often under a significant financial strain, which can lead to job instability and under-staffing [2].

Several studies conducted in the UK, USA, and Europe have reported levels of burnout among psychiatrists [21]. The personnel is exposed to specific stressors, such as taking care of patients suffering from mental disorders, dealing with the violent patient together with the environment, low support at work, complicated relationships with the health professionals, overwork, inadequate resources and organization, threats to self-esteem and personal threats [22].

Also, psychiatrists report a range of personal stressors in their work, and one personal stress, which psychiatrists find challenging to cope with is patient suicide [21].

Furthermore, nowadays, burnout is prevalent throughout the process of medical training [17, 23-25]. Medical students and trainees have higher levels of stress and burnout compared with the general population [25]. Medical residents, especially psychiatry residents, are affected by two significant issues – stress and burnout [25]. Burnout can challenge residents’ professional development, place patients at risk, have a significant influence on a variety of personal costs, and may lead to various mental health problems that need to be addressed, including substance abuse and suicidal ideation [23, 25].

Another variable causing burnout is the fact that many medical students can have burnout symptoms while attending medical school (academic burnout) and may even start their career as residents already having burnout symptoms [26].

However, burnout is harmful not only to clinicians [27]. There are negative consequences and negative effects on patient care, professionalism, physicians’ care and safety, and the viability of healthcare systems [14]. Even more, there are studies that emphasize that burnout has a negative impact on the patient’s care and satisfaction [27]. If left unattended, burnout can have unwanted costs, including disruption to work, reduced productivity, decreased job satisfaction, decreased quality of patient care, disruption of personal relationships, and increased anxiety and depression [5].

Moreover, during residency, stress sources can be divided into three components:

1. situational (workload, sleep deprivation, and poor learning environment);
2. personal (family, isolation, and financial);
3. professional (overwhelming patient responsibility and information) [28].

In addition, psychiatry residency training is a transitional period, and burnout among residents is associated with reduced empathy and problematic strategies for coping with patient-related stress. On the other hand, among residents from different medical specialties, 40% of the psychiatry residents endorse burnout – less than other specialties but more compared to family physicians [17].

A systematic review found that there are three groups of factors studied in relationship to burnout among psychiatric residents: (1) demographic factors, (2) training and work-related factors, (3) learner factors. According to this, burnout in psychiatric residents was associated with specific demographic (non-parental status), training (juniors years of training, lower priority of psychiatry as career choice, lack of clinical supervision, discontinuation from training), work (high workload, long work hours, deficient rest) and learner factors (more stressors, higher anxiety, depressive symptoms, low levels of self-efficacy, decreased empathic capacity, poor coping, self-medication and use of mental health services) [15].

In Romania, the study of burnout is in its early stages. There is no specific data for the mental health sector, and there are no economic resources for its research [29].

Given its prevalence and the severe nature of the consequences, taking action is needed to prevent burnout among doctors in training and helping those who are struggling to get the necessary care [23]. However, there is scarce literature connecting burnout and psychiatry. Moreover, until the present, we have not found any research conducted on Romanian residents on burnout using the OLBI instrument. Therefore, the present study was designed to determine the prevalence and associated features of burnout among Romanian psychiatry residents. More precisely, the objectives were the following:

• To highlight the prevalence of burnout on three levels (low, moderate, high) using the OLBI instrument;
• To determine the socio-demographic profile of the Romanian psychiatric residents in association with high burnout levels;
• To investigate the impact of socio-demographic factors on the high burnout levels in Romanian psychiatric residents.

## Literature review

Burnout is defined and measured as a work-related syndrome that develops progressively as an individual response to particular work-related events [26, 27].

Even if there are more than a few models regarding the burnout concept, experts generally emphasize that burnout covers two main symptoms: high levels of exhaustion and a distant/cynical attitude towards work [30].

On the one hand, burnout was initially described as a complex concept characterized by various degrees of emotional exhaustion, depersonalization (i.e., feeling detached from or callous toward patients) and a low sense of personal accomplishment [31], and, on the other hand, definitions emphasize that burnout should be conceptualized as a primarily work-related syndrome of (at least) exhaustion and depersonalization/cynicism. Further, previous findings underlined that exhaustion and cynicism might be considered the core symptoms of burnout, whereas personal accomplishment might instead be interpreted as a consequence of burnout [32].

The most frequently known features responsible for burnout are classified as being either organizational or individual. Organizational features are excessive workload, long working hours, or low perceived social support within the organization, and individual features refer to individual characteristics of adapting to challenging circumstances, such as inefficient coping strategies and alexithymia [29]. Nevertheless, physicians were reported to use adaptive coping methods while facing burnout [29].

From a psychiatric perspective, burnout is not included in the Diagnostic and Statistical Manual of Mental Disorders (DSM), Fifth Edition [33], but it was included in the International Classification of Diseases (ICD), 11th Revision [34]. It was also included in ICD-10, among factors that influence individual wellbeing (Z73.0), but the definition is more detailed in ICD-11 [35]. According to ICD-11, burnout is a syndrome theorized as a consequence of chronic workplace stress that has not been successfully managed, and it was defined by three dimensions: (1) feelings of energy depletion or exhaustion; (2) increased mental distance from one’s job, or feelings of negativism or cynicism related to one’s job; and (3) reduced professional efficacy [34].

Although Maslach Burnout Inventory – General Survey (MBI-GS) is considered “the gold standard” for burnout measurement concerning occupational stress, OLBI is a reliable and valid measurement instrument for the assessment of burnout, which can be used as an alternative to the widely used MBI [26]. There is a study which emphasizes that burnout, as measured by the MBI, consists of theoretically distinct aspects - an individual state (emotional exhaustion), a coping strategy (depersonalization) and a consequence (reduced personal accomplishment) - that should be studied in their own right, instead of lumping them together under the label of burnout [36].

Undoubtedly, preceding research has shown that a lack of personal accomplishment works differently from the two other MBI dimensions, suggesting that lack of personal accomplishment might not be part of the burnout syndrome [32].

Besides, there are studies that emphasize that depersonalization is a coping strategy developed in particular circumstances and should be better analyzed as such, together with other coping strategies and in the light of the complete literature on coping and stress. The same logic applies with regard to the (feeling of) reduced personal accomplishment: Low accomplishment should be perceived as one of many consequences of long-term stress [36]. Based on this criticism, we decided to use the Oldenburg Burnout Inventory as an alternative instrument to measure burnout.OLBI was initially developed in order to overcome most of the MBI-GS limitations for the reason that some researchers have criticized the psychometric qualities of MBI-GS, pointing out that it measures only affective exhaustion, that it includes the sub-dimension of professional efficacy and the wording of its items is one-directional [26].

OLBI has two subscales exhaustion and disengagement (from work) [10]:

• Exhaustion is defined as a consequence of intense physical, affective, and cognitive strain, for example, a long-term consequence of prolonged exposure to specific job demands.
• Disengagement is related to distancing oneself from ones’ work in general, work object, work content. Additionally, the disengagement items concern the relationship between employees and their jobs, particularly concerning the identification with work and willingness to continue in the same occupation. Disengaged employees endorse negative attitudes toward their work objects, work content, or work in general.

Moreover, OLBI can be used in order to measure burnout (with its dimensions) and work engagement as bipolar constructs [31]. In addition, it provides high scale reliability (Cronbach’s alpha=0.63) as well as on its subscales, exhaustion (Cronbach’s alpha=0.87) and disengagement (Cronbach’s alpha=0.81) [26].

## Material and Methods

### Participants

Participants were psychiatric residents employed in different public institutions in Romania. Inclusion criteria covered psychiatric residents, with more than one year of work experience, who agreed to fill in our questionnaire. Out of 150 psychiatric residents, 116 (77.33%) agreed to join this study by completing an informed consent form. Out of 116 participants, 89 (76.7%) were females, and 27 (23.3%) were males, all being from urban residential areas. The mean age was 28.03 (±2.99). 72.4% of the residents worked in mono-specialty Psychiatric Hospitals. Further, 89.7% of them were satisfied with their salaries, 85.3% worked night shifts, and 94% did not consider having the necessary resources (time, logistics) to attend their patients. Additionally, the mean number of night shifts per month was 1 (±0.611), and the mean number of attended patients per day was 7.09 (±3.87), ranging from 2 to 20.

### Procedure

The design of the study was cross-sectional. Confidentiality and anonymity were guaranteed. As such, all participants received a questionnaire that comprised two sections: the first section gathered exclusively socio-demographic information about the participants: age, gender (male/female), workplace, residential area (urban/rural), the satisfaction of the respondents with their salaries (yes/no), the number of night shifts worked per month, if the respondents considering they had enough resources to attend their patients (yes/no); the second section consisted of the OLBI scale for determining the respondents’ burnout level.

OLBI has two dimensions (exhaustion and disengagement from work) evaluated by 16 items: 8 items measure the exhaustion, and 8 items measure disengagement from work. Both dimensions were evaluated by four positively worded items and four negatively worded items. Items were scored by using a scale ranging from 1 to 4 (Strongly agree – Strongly disagree) [26, 31].

### Analysis

Data were collected and processed using the SPSS Statistics 20.0 software package. In order to assess the proposed objectives, the data process was comprised of two stages. As such, the first stage of the analysis included a sample description, using measures that were specific to both quantitative variables such as the mean and standard deviation, as well as in terms of frequency for qualitative variables.

Threshold values for the classification of burnout into “high”, “moderate”, and “low” levels were calculated by splitting the scores into thirds. The second stage of the analysis was comprised of multiple linear regression with the purpose of investigating the associations between the socio-demographic variables and the predicted variable, namely burnout. The threshold for statistical significance in all tests was p<0.05

## Results

### a. The psychometric validation of the OLBI scale was conducted using two methods: Cronbach’s alpha coefficient, which should be higher than 0.80, and the factor analysis.

The Cronbach’s alpha coefficient pointed out a value of 0.814 (Table 1) and the factorial analysis revealed that the two components, Exhaustion and Disengagement, loaded on one factor, Burnout (Table 2).

**Table 1:** The Cronbach’s alpha coefficient value of the OLBI scale.

**Item-Total Statistics**	**Scale Mean if Item Deleted**	**Scale Variance if Item Deleted**	**Corrected Item-Total Correlation**	**Cronbach’s alpha**
Exhaustion	27.64	28.894	0.686	0.814
Disengagement	25.01	29.817	0.686	-

**Table 2:** The factorial analysis of the OLBI scale.

Component Matrix	Component
	1
Exhaustion	0.918
Disengagement	0.932

### b. The socio-demographic profile of psychiatric residents with high burnout levels

Twenty-six psychiatric residents suffered from high levels of burnout (Table 3). The vast majority of respondents were females (84.6%) with a mean age of 27.35 (±3.81). Moreover, all participants with high burnout were satisfied with their salary and their work but dissatisfied with the resources available for attending patients. Most of the respondents worked night shifts (80.8%) and with a mean monthly shift of 1 (±0.49), the mean number of patients being attended per day was 6.77 (±2.99), ranging from 2 to 15 patients.

**Table 3:** The socio-demographic profile of the psychiatric residents with high levels of burnout.

Variable	Frequency	Percentage
Gender		
Female	22	84.6
Male	4	15.4
Mean age	27.35±3.81	
Residence area		
Urban	26	100
Satisfaction with monthly salary		
Yes	26	100
Satisfaction with work		
Yes	26	100
Night shifts		
Yes	21	80.8
No	5	19.2
Mean of monthly night shifts	1.00±0.49	
Satisfaction with available resources to attend patients		
No	26	100
Mean number of patients being attended per day	6.77±2.99	

### c. The prevalence of burnout in psychiatric residents

The data collection was divided into percentiles of 25.50 and 75. The returned thresholds for the total OLBI scale were 44.54, and 59, respectively. As such, the burnout scores were classified into low (25.9%), moderate (51.7%), and high (22.4%) (Table 4).

**Table 4:** Burnout levels.

**Level**	**Range scores**	**Frequency**	**Percentage**
**Low**	<44	30	25.9
**Moderate**	44-59	60	51.7
**High**	>59	26	22.4

Table 5 reveals that 23.3% of the respondents (n=27) were high in Exhaustion and 25% (n=29) were high in Disengagement.

**Table 5:** The Burnout levels on components.

Burnout component	Level	Range scores	Frequency	Percentage
**Exhaustion**	Low	<21	34	29.3
	Moderate	21-29	55	47.4
	High	>29	27	23.3
**Disengagement**	Low	<24	33	28.4
	Moderate	24-31	54	46.6
	High	>31	29	25.0

### d. The impact of socio-demographic factors on burnout

Satisfaction with work (beta=0.253, p< 0.005) and age (beta=-0.236, p<0.009) explained 10% of the variance in the Total OLBI Burnout. In other words, a unit maximization in burnout is proportional to a unit maximization in satisfaction with work. However, a unit maximization in burnout is a minimization in the age of the respondents, suggesting that the younger the respondents are, the less they feel burned out. Moreover, on OLBI components, on one hand, Exhaustion was explained by none of the socio-demographic characteristics of respondents and on the other hand, 21% of the variance in Disengagement was explained by satisfaction with work (beta=0.357, p<0.001), age (beta=-0.275, p<0.002) and gender (beta=-0.199, p<0.021). More precisely, a unit maximization in the burnout score, leads to an increase in the satisfaction with work, meaning the more the psychiatry residents feel disengaged, the more burned out they will be. Further, a unit maximization in burnout will trigger a decrease in age, suggesting that younger residents feel less disengaged, and the male residents tend to be more disengaged.

## Discussion

The current study showed that out of 116 psychiatric residents, the vast majority - 51.7% (n=60) experienced moderate burnout, and only 22.4% (n=26) experienced high burnout. This could be a result of the fact that, as stated in previous research studies, from all physicians who might experience burnout, psychiatrists are most likely to search for help, while other physicians might consider that seeking for help could have severe consequences on their career [1]. From another perspective, only 25.9% (n=30) experienced low burnout. More than that, a range of specific coping interventions are available and consist of mindfulness techniques, stress-reduction exercises, and overall efforts to support self-care [27]. In a systematic review and meta-analysis of interventions to address physician burnout, the majority focused on individual interventions, and emotional exhaustion and depersonalization were noted to be useful in dropping burnout [14]. Undesirably, some coping strategies may have short-term effects but can have long-term negative costs. These unhealthy coping approaches include denial, depersonalization, compartmentalization, suppression, social isolation, and substance abuse [27]. Regarding coping strategies, there are data which reported that Romanian physicians use recurrently dysfunctional coping strategies (e.g., denial, substance use, behavioral disengagement) [11].

Furthermore, all participants with high burnout (n=26) were satisfied with their salary and their work but dissatisfied with the available resources for attending patients. The Romanian policy of increasing incomes for medical personnel (physicians, residents, and nurses) might have had an essential influence on our results. Residency is an appropriate period for implementing prevention, supervisory, and self-care strategy. During this stage, minimizing burnout might be possible by reducing personal vulnerability to burnout by implementing particular strategies.

Several limits need to be addressed regarding this study. First, our research had a small number of participants. Second, burnout is a dynamic process, and results may be different if the assessments are conducted in a different period or on multiple occasions.

## Conclusion

In conclusion, adding stress management training to the medical education curriculum could help the residents to deal more effectively with the training strain, develop personal techniques for helping themselves to improve their professional path, and potentially prevent upcoming physician burnout [28].

## Conflict of Interest

The authors confirm that there are no conflicts of interest.
